# Temperament Dimensions and Awakening Cortisol Levels in Attention-Deficit/Hyperactivity Disorder

**DOI:** 10.3389/fpsyt.2022.803001

**Published:** 2022-04-25

**Authors:** Alessandra Carta, Isabella Vainieri, Anna-Sophie Rommel, Alessandro Zuddas, Jonna Kuntsi, Stefano Sotgiu, Nicoletta Adamo

**Affiliations:** ^1^Child Neuropsychiatry Unit, Department of Medical, Surgical and Experimental Sciences, University of Sassari, Sassari, Italy; ^2^Social, Genetic and Developmental Psychiatry Centre, Institute of Psychiatry, Psychology and Neuroscience, King's College London, London, United Kingdom; ^3^South London and Maudsley National Health Service Foundation Trust, London, United Kingdom; ^4^Department of Psychiatry, Icahn School of Medicine at Mount Sinai, New York, NY, United States; ^5^Department of Biomedical Sciences, Section of Neuroscience and Clinical Pharmacology, University of Cagliari, Cagliari, Italy; ^6^Child and Adolescent Neuropsychiatry Unit, ‘A.Cao', Paediatric Hospital, ‘G. Brotzu' Hospital Trust, Cagliari, Italy

**Keywords:** ADHD, temperament, cortisol, arousal, biomarkers

## Abstract

**Objective:**

To investigate whether temperament dimensions, Effortful Control (EC), Surgency-Extraversion (SE), and Negative Affectivity (NA), are associated with attention-deficit/hyperactivity disorder (ADHD) and how they relate to awakening cortisol levels, as a proxy measure of peripheral arousal.

**Methods:**

Parent-rated temperament and saliva samples were collected from 55 children with ADHD and 65 age-matched controls.

**Results:**

Compared to controls, youths with ADHD showed lower EC, higher NA, and lower awakening cortisol levels but did not differ in SE. Similar findings emerged in dimensional analyses linking temperament traits to inattention and hyperactivity-impulsivity symptoms. The results remained unchanged when controlling for the presence of co-occurring opposition-defiance and anxiety traits, as well as medication status. Temperament dimensions were not associated with cortisol levels.

**Conclusions:**

Poor temperamental emotional and cognitive self-regulation showed significant associations with ADHD but did not appear to be linked to the under-arousal typically seen in ADHD.

## Introduction

Temperament is conceptualized as a broad dimension of personality that emerges in childhood and broadly relates to differences in emotional reactivity and behavioral and cognitive self-regulation (1). Furthermore, these temperament dispositions in childhood are thought to have physiological substrates (2, 3) and play a role in different developmental outcomes (4). In particular, research has established links between temperament profiles and neurodevelopmental disorders such as attention deficit hyperactivity disorder (ADHD) (5, 6), but the underlying neurobiological mechanisms of such associations remain understudied.

Studies on parent-rated temperament questionnaire data have investigated how the three broad temperament dimensions, effortful control, negative affectivity, and surgency-extraversion relate to ADHD as a diagnosis and a continuum of symptom scores. Compared to typically developing individuals, children and young people with ADHD have been found to have low effortful control (i.e., less efficient voluntary self-regulation of attention and behavior), elevated negative affectivity (i.e., high levels of negative emotional reactivity, poor coping, and self-soothability), and increased surgency-extraversion (i.e., positive affect and responsiveness to stimulation and novelty) (6–8). Dimensional analyses also found a relationship between low effortful control traits and inattentive and hyperactivity-impulsivity symptoms (9–16). In contrast, high levels of the surgency-extraversion temperamental trait are most commonly associated with hyperactive-impulsive symptoms (12).

Beyond ADHD, there is evidence that other psychiatric conditions are also related to temperament dimensions. For example, disruptive behavior disorders, which often co-occur with ADHD, including the diagnosis of oppositional-defiant disorder (ODD), are also related to temperamental negative affectivity (6, 12, 17, 18). Additionally, anxiety symptoms are associated with high negative affectivity and low effortful control (6, 19, 20). Thus, studies have investigated whether comorbid traits may affect the association between temperament traits and ADHD. Further, the associations between negative affectivity and ADHD diagnosis or traits may be observed only in the presence of anxiety (20) or oppositional-defiant symptoms (12).

Temperament subtypes have previously been linked to the peripheral physiological measures of the autonomic nervous system. Specifically, a recent study demonstrated a significant association between negative affectivity and reduced parasympathetic response, while high surgency-extraversion was linked to sympathetic responses (2). Another candidate for investigating the potential links between physiological measures and temperament in ADHD is cortisol, a peripheral measure of arousal. Baseline cortisol, commonly measured with awakening values of this hormone, is hypothesized to be a biological proxy measure of behavioral self-regulatory systems, activity level, cognition, and under-arousal in ADHD (13, 21–25). Studies have found low awakening cortisol levels concerning ADHD diagnosis and symptoms. However, this evidence is currently inconsistent (26), with some studies showing reduced cortisol levels in ADHD (27–29) and others showing no associations with ADHD traits (30) or case-control differences (31) requiring further replication. Additionally, only children with ADHD and ODD, but not those without comorbid disruptive behavior disorders, showed lower awakening cortisol levels than controls in an earlier study (32). Furthermore, no studies to date have tested whether awakening cortisol may explain the link between temperament and ADHD.

Nevertheless, there is evidence that pharmacological treatment may impact the observed atypical cortisol levels in ADHD. Some authors have reported higher awakening cortisol levels in medicated children with ADHD than controls (33, 34). Conversely, other investigators did not find significant differences between medicated participants with ADHD and controls (35, 36). Finally, despite an increase in cortisol levels after 1 month of treatment with methylphenidate, one study found that cortisol levels gradually decreased and returned to baseline levels at six or more months after treatment initiation (37). It remains unclear whether awakening cortisol levels differ in participants with ADHD when they withdraw from their treatment after taking pharmacological treatment with ADHD medications.

The present study aimed to examine the association of ADHD with temperament dimensions and low cortisol levels and test the links between temperament and cortisol. Specifically, we first investigated whether temperament dimensions and awakening cortisol levels differ between children with ADHD and controls, including their correlation with ADHD symptoms (inattention and hyperactivity-impulsivity). Second, we examined whether the three temperament dimensions were associated with awakening cortisol levels in the ADHD and control groups. Third, we tested whether oppositional-defiant and anxiety traits also relate to temperament dimensions and cortisol levels in each group and whether the association of ADHD with each temperament or cortisol measure remains when controlling for these comorbid psychiatric traits. Finally, previous evidence suggests that methylphenidate may affect the awakening cortisol levels in individuals with ADHD. The fourth aim is to examine whether cortisol levels change after a medication washout in a subsample of participants with ADHD who were undergoing treatment with methylphenidate.

## Materials and Methods

All procedures performed in this study involving human participants were per the ethical standards of the institutional and national research committee and with the 1964 Declaration of Helsinki and its later amendments or comparable ethical standards. Our study was registered with ClinicalTrials.gov (Identifier: NCT04326543) on 25 March 2020.

### Participants

For this study, we enrolled sixty-seven individuals with ADHD and seventy typically developing youth (henceforth, controls) aged between 4 and 16 years. Individuals with ADHD were recruited and assessed from the Child and Adolescent Neuropsychiatry Unit, University of Sassari, Italy. The assessment was completed as part of their routine psychiatric assessment using an unstructured clinical interview with the participant's parents or legal guardians led by a child and adolescent psychiatrist, as well as screening for ADHD and other psychiatric symptoms through standardized questionnaires and direct observations with the participants in the assessment appointment, which led to a DSM-5-based diagnosis of ADHD.

Exclusion criteria were IQ below seventy, neurological disorders (e.g., epilepsy, gravis myasthenia), genetic (e.g., neurofibromatosis type 1, X-fragile, and Down syndrome), and medical conditions mimicking ADHD symptoms (e.g., hyperthyroidism as well as a diagnosis of bipolar disorder, major depressive disorder, post-traumatic stress disorders or psychosis. Ongoing treatment with psychotropic agents other than ADHD medications was also exclusionary. Controls were recruited from pre-school, primary, and secondary schools in the local catchment area. Inclusion as controls was determined by an unstructured interview conducted with the parents and teachers by the lead author, child, and adolescent psychiatrist. Controls were excluded if they presented with any learning difficulties reported in the parent and teacher interviews, including mild, moderate, or severe symptoms of ADHD in the clinician-led rating scale (see below). Signed informed consent was obtained from parents, and participants aged ≥12 years signed a written assent, as approved by the ethical review board of the University of Sassari. Data on temperament and cortisol were collected between 1 January and 1 June 2017. Complete data on ADHD symptoms, temperament ratings, and basal awaking salivary cortisol samples were available for 55 individuals with ADHD and 65 controls, who constituted the final sample. A total of 33 youths with ADHD were undergoing treatment with methylphenidate for at least 6 months at the time of assessment; the remaining participants were medication-naïve.

In the ADHD group, 29 participants were receiving cognitive behavioral therapy targeting the ADHD symptoms as well as the opposition-defiant or anxiety comorbid traits.

Participants in both the ADHD and control groups were not compensated for their participation in the study.

### Measures

#### ADHD Traits

The presence or absence of ADHD symptoms in all participants was ascertained by administering the clinician-led Swanson, Nolan, and Pelham Rating Scale-IV (SNAP-IV) (38, 39). The SNAP-IV consists of 26 items that are rated on a 4-point scale (not at all, just a little, quite a bit, very much). The items are divided between three subscales: inattention (nine items), hyperactivity/impulsivity (nine items), and oppositional (eight items). Subscale scores are calculated by creating an average. Here, we used the scores from the inattention and hyperactivity/impulsivity subscales, with a total of 18 items. These inattention and hyperactivity/impulsivity items can be combined to create a combined ADHD score (38). Higher scores represent more problem symptoms. In our study, the SNAP-IV was administered to parents by a clinician. Research has demonstrated criterion validity of the SNAP-IV, showing that children with higher ratings on the SNAP-IV were more likely to receive a DSM-5 diagnosis of ADHD (40).

#### Temperament Dimensions

Parents of all participants completed the Italian version of Mary Rothbart's Temperament Questionnaire (41); (https://research.bowdoin.edu/rothbart-temperament-questionnaires/instrument-descriptions/the-early-adolescent-temperament-questionnaire/), a caregiver's rated measure designed to provide a detailed assessment of temperament. We used the Children Behavior Questionnaire (CBQ) (41) for children aged 4–7 years. For those aged 7–10 years, we administered the Temperament in Middle Childhood Questionnaire (TMCQ) (42). Finally, the Early Adolescents' Temperament Questionnaire (EATQ) (43) was used for youths aged 11–16 years. For each item, scores ranged from 1 to 7 in the CBQ and 1 to 5 in the TMCQ and EATQ. Effortful control (EC), negative affect (NA), and surgency-extraversion (SE) were measured for each participant using the scales suggested by Rothbart et al. (41). Composite scale scores were generated by reverse-scoring the selected items and computing the average score. The reliability of the scales for measuring temperament dimensions has been previously demonstrated (2, 44, 45).

#### Awakening Cortisol Levels

Cortisol levels were measured in saliva samples. Salivary samples were collected on a weekday between 7 and 8 a.m., within 60 min of awakening. Sampling was performed at the unit's outpatient department for participants with ADHD during a routine review appointment and school for controls. Participants were instructed to sample their saliva before tooth brushing, at least 30 min after eating, drinking, chewing gum, or smoking. Saliva samples (5 ml) were collected in large Falcon test tubes and stored in a −20°C medical freezer until completion of the study when they were assayed for cortisol. Salivary samples were analyzed in a single batch using a high-sensitivity immuno-electro-chemo-luminescence assay (ECLIA) at the Laboratory of Medicine at San Raffaele Hospital, Milano Italy. The lower limit of detectable sensitivity was 0.54 μg/l. The samples of five controls and twelve youths with ADHD could not be obtained or had insufficient saliva volume to test for cortisol. Therefore, cortisol concentrations were provided in μg/l and converted to nmol/l. For individuals with ADHD taking methylphenidate at study entry (*N* = 33), the sample was collected twice: during their ongoing treatment for at least 6 months and after 4 days from withdrawing their medication.

#### Oppositional-Defiant and Anxiety Traits

The Long Version of Conners' Parents Rating Scale (CPRS-R:L) (46) was used to estimate levels of oppositional-defiant and anxiety traits.

CPRS were available for 53 participants with ADHD (96%) and 54 controls (83%). Oppositional defiant traits were measured using the 10 items of the Oppositional scale (labeled as scale A). Further, anxiety traits were measured using the eight items of the Anxiety-shyness scale (labeled as scale D) of the CPRS-R:L. Results reported herein are total scores for these two scales.

When calculating indices of reliability for each scale (see Supplementary Table S1), the *Cronbach's Alpha* reliability test was estimated as (α = 0.65 and 0.66) for the opposition-defiance and the anxiety scale, respectively. While in line with the interpretation of George and Mallery (47) an alpha above 0.6 is questionable, an alpha between 0.5 and 0.7 has been deemed to reflect moderate reliability by others (48). In line with Nunally and Bernstein (49), we therefore considered these as acceptable indices for the purpose of this exploratory research on the relationship of ADHD and comorbid psychiatric traits with temperament dimensions and cortisol.

### Statistical Analyses

Analyses were performed using the R software (version R 3.4.1). Analysis of variance covarying for sex (ANCOVA) compared the two groups on the three temperament dimensions and the awakening cortisol levels. A series of linear regression model analyses were used to test the association of temperament dimensions and cortisol levels with ADHD traits and the oppositional-defiant and anxiety traits in the ADHD and control groups. Specifically, we modeled each temperament and cortisol measure as a function of the participant's ADHD, oppositional-defiant, and anxiety symptom scores. When a significant association emerged for the examined measures both with ADHD traits and with either comorbid trait, we repeated the analysis testing the relationship of temperament and cortisol with ADHD by controlling for the effects of oppositional-defiant or anxiety traits. We performed the repeated-measures analysis of variance to test the difference between cortisol levels on- and off-methylphenidate treatment in participants with ADHD. Before analysis, cortisol levels were log-transformed to normal levels. Analyses were then performed using standardized scores for all the measures. Because the dimensional analyses were carried out using standardized scores, the β coefficients resulting from the regression models represent a standardized effect size measure. In particular, a 1–standard deviation change in each temperament dimension leads to β change in the ADHD symptoms, cortisol, and opposition-defiance/anxiety traits. The effect size was comparable to that of the correlation coefficients.

We calculated a *post-hoc* power analysis for the differences between the ADHD and control groups for all endpoints, using a probability of type I error of 0.05. Comparisons for scores of core ADHD symptoms and comorbid traits were adequately powered, with a statistical power >95%. Comparisons for temperament and basal cortisol levels were also well-powered, reaching 90% power, except for the surgency-extraversion temperament, whose statistical power was lower (77.5%). For variables with high missing data, the power is reduced. The study addressed five specific and pre-planned research questions, which led our analyses toward well-defined hypotheses, based on previous reference literature. Therefore, no multiple testing correction was conducted, in line with previous guidelines (50).

## Results

### Demographics

The ADHD and control groups were matched for age but differed significantly in sex distributions (Table 1). All participants were White. As expected, ratings of all ADHD symptom scores were significantly elevated in children and adolescents with ADHD relative to control participants, both in inattentive and hyperactive-impulsive symptoms (Table 1). Additionally, levels of oppositional-defiant and anxiety traits were significantly greater in the ADHD group than in the control group (Table 1).

**Table 1:** Sample characteristics.

	**ADHD**		**Controls**		**Group comparisons**		
	***n*** **=** **55**		***n*** **=** **65**		***X^**2**^***	**df**	***p***
	***n***	**%**	***n***	**%**			
Males	48	87.27	30	46.15	20.37	1, 118	<0.001
	**ADHD**		**Controls**		**ANOVA**		
	**Mean**	**SD**	**Mean**	**SD**	**F**	**df**	***p***
Age (years)	9.47	3.12	10.01	3.00	0.94	1, 118	0.34
**ADHD traits**							
Inattention	21.92	3.62	4.81	4.37	532.4	1, 118	<0.001
Hyperactivity-impulsivity	22.29	4.70	3.64	3.95	556.9	1, 118	<0.001
**Associated psychiatric traits**							
Oppositional-defiant disorder	74.03	15.69	44.57	7.49	154.5	1, 105	<0.001
Anxiety	58.77	14.46	44.88	6.92	40.33	1, 105	<0.001

*ADHD, attention deficit hyperactivity disorder; SD, standard deviation. Associated psychiatric traits were evaluated using the Long Version of Conners' Parents Rating Scale (CPRS: RL) available for fifty-three participants with ADHD and fifty-four controls*.

Participants from three age groups were included in our study: early childhood (4–6 years), middle childhood (7–10 years) and adolescence (11–16 years). The ADHD group consisted of eleven 4 to 6-year olds (20.0%), twenty-five 7- to 10-year olds (45.5%), and nineteen 11- to 16-year olds (34.5%). The control group consisted of fifteen 4- to−6-year olds (23.4%), twenty-nine 7- to 10-year olds (45.3%), and twenty 11- to 16-year olds (31.3%).

### Do Temperament Dimensions and Awakening Cortisol Levels Differ Between Individuals With ADHD and Controls?

**Figure 1:**
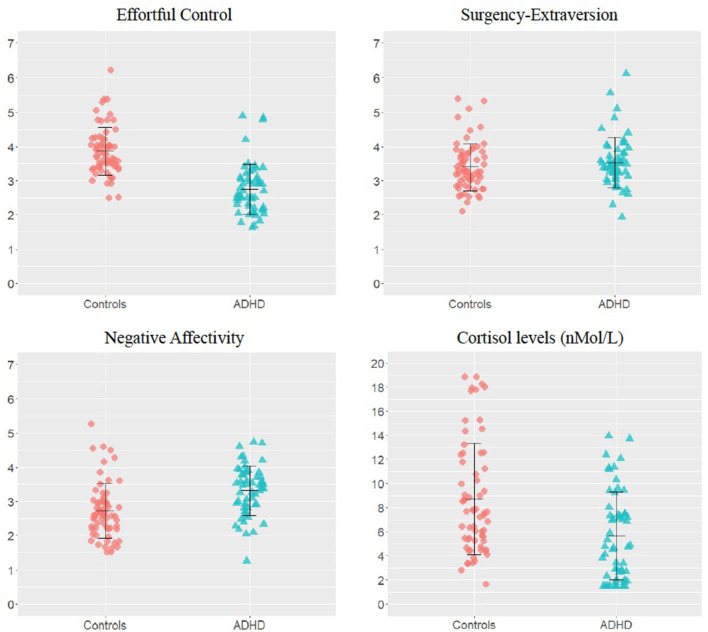
Group differences on temperament dimensions and awakening cortisol levels. Unstandardized scores on the temperament dimensions subscales and awakening cortisol levels in the attention-deficit/hyperactivity disorder (ADHD) and control groups. The error bars represent standard deviations.

Unstandardised temperament dimension scores and awakening cortisol levels are shown in Figure 1. Participants with ADHD showed significantly lower EC and higher NA than controls [EC: *F*_(1,117)_ = 76.03; *p* < 0.001; NA: *F*_(1,117)_ = 31.20; *p* < 0.001], whereas SE scores did not differ across groups [*F*_(1,117)_ = 0.71; *p* = 0.40] (Figure 1). Group comparisons also showed that the awakening cortisol levels were significantly lower in the ADHD group than in the control group [*F*_(1,117)_ = 13.52; *p* < 0.001; Figure 1].

### Are Temperament Dimensions and Awakening Cortisol Levels Associated With the Two ADHD Symptom Dimensions (Inattention and Hyperactivity-Impulsivity)?

Inattention showed a significant negative association with EC scores in both the control and ADHD groups (Table 2). The inattention × group interaction for EC did not reach statistical significance (β = 0.40, 95% CI = −0.17 to 0.97, *p* = 0.17), indicating that the association of inattention with EC did not differ between groups. A significant association emerged between hyperactivity-impulsivity and low EC scores in the controls (Table 2). Although of small effect size, this association did not reach statistical significance in the participants with ADHD (Table 2). Further, a significant positive hyperactivity-impulsivity × group interaction for EC (β = 0.78, 95% CI = 0.22–1.34, *p* < 0.01) indicated a significantly smaller association of hyperactivity-impulsivity with EC in the ADHD group compared to the controls. The relationship between inattention and NA had a small effect size in controls and a moderate effect size in the ADHD group. Nevertheless, it only reached a trend for statistical significance in both groups (Table 2). Hyperactivity-impulsivity significantly predicted NA scores in the controls, but not in the ADHD group (Table 2), and the interaction of hyperactivity-impulsivity with group was not significant (β = −0.35, 95% CI = −1.10 to 0.38, *p* = 0.35). Small effect-size associations of inattention with SE in controls and of hyperactivity-impulsivity with SE in participants with ADHD did not reach statistical significance (Table 2); no other significant associations or trait × group interactions emerged. No significant associations emerged between inattention or hyperactivity-impulsivity and cortisol levels in the control and ADHD groups (Table 2).

**Table 2:** Association of each subdomain of the ADHD traits with the three temperament dimensions and awakening cortisol levels.

	**Inattention**		**Hyperactivity-impulsivity**	
	**Controls**	**ADHD**	**Controls**	**ADHD**
	**β (95% CI)**	**β (95% CI)**	**β (95%, CI)**	**β (95% CI)**
Effortful control	−0.87 (−1.22, −0.52)^***^	−0.47 (−0.93, −0.01)^*^	−1.10 (−1.52, −0.69)^***^	−0.32 (−0.70, 0.05)∧
Negative affectivity	0.39 (−0.07, 0.86)∧	0.60 (−0.01, 1.20)∧	0.70 (0.15, 1.25)^*^	0.34 (−0.16, 0.84)
Surgency-extraversion	0.45 (−0.06, 0.96)∧	0.30 (−0.37, 0.97)	0.29 (−0.32, 0.90)	0.50 (−0.05, 1.05)∧
Cortisol	0.33 (−0.17, 0.84)	0.02 (−0.64, 0.68)	0.18 (−0.42, 0.78)	−0.48 (−1.02, 0.06)∧

*Results from the final regression model examining associations of inattention and hyperactivity-impulsivity symptoms with the three temperamental dimensions and with awakening cortisol in the ADHD and control groups, controlling for sex. ADHD, attention deficit hyperactivity disorder; CI, confidence interval fixed at 95%, ranged between 2.5 and 97.5%*.

∧ *p < 0.1*;

* *p < 0.05*;

*** *p < 0.001*.

### Are Awakening Cortisol Levels Associated With Any of the Three Temperament Dimensions?

In both the ADHD and control groups, we found no significant associations between cortisol levels and EC (controls: β = −0.06, 95% CI = −0.39 to 0.25, *p* = 0.68; ADHD: β = −0.08, 95% CI = −0.44 to 0.27, *p* = 0.64), NA (controls: β = −0.15, 95% CI = −0.45 to 0.13, *p* = 0.28; ADHD: β = −0.15, 95% CI = −0.45 to 0.13, *p* = 0.28), or SE (controls: β = 0.02, 95% CI = −0.21 to 0.25, *p* = 0.84; ADHD: β = < 0.01, 95% CI = −0.26 to 0.26, *p* = 0.99).

### Are Oppositional-Defiant and Anxiety Traits Associated With Temperament and Cortisol Levels, and Do They Affect the Relationship Between ADHD and Temperament?

No significant associations emerged for either oppositional-defiant or anxiety traits with EC, SE, or cortisol levels. Further, we did not find substantial trait × group interactions for either opposition-defiance or anxiety on any of these temperament dimensions or cortisol levels (Table 3). We found significant associations of opposition-defiance and anxiety with NA in the ADHD group but not in the controls (Table 3). Despite a greater magnitude of the regression parameters in the ADHD group than in controls for these associations, opposition-defiance and anxiety traits did not show significant interactions with the group (Table 3).

**Table 3:** Results of regression model analyses showing the association of oppositional-defiant and anxiety traits with the three temperament dimensions and awakening cortisol levels, as well as interaction effects of these psychiatric traits with diagnostic group.

	**Oppositional-defiant traits**			**Anxiety traits**		
	**Controls**	**ADHD**	**Group × trait**	**Controls**	**ADHD**	**Group × trait**
	**β (95% CI)**	**β (95% CI)**	**β (95% CI)**	**β (95%, CI)**	**β (95% CI)**	**β (95% CI)**
Effortful control	−0.25 (−0.73, 0.24)	−0.15 (−0.38, 0.08)	0.09 (−0.44, 0.64)	−0.05 (−0.42, 0.31)	−0.02 (−0.20, 0.16)	0.03 (−0.38, 0.44)
Negative affectivity	<0.01 (−0.59, 0.59)	0.54 (0.26, 0.82)^**^	0.54 (−0.12, 1.20)	<0.01 (−0.46, 0.46)	0.33 (0.11, 0.55)^**^	0.32 (−0.18, 0.83)
Surgency-extraversion	0.43 (−0.22, 1.09)	0.01 (−0.31, 0.32)	−0.43 (−1.15, 0.30)	−0.48 (−0.96, <0.01)∧	−0.12 (−0.36, 0.11)	0.36 (−0.18, 0.89)
Cortisol	0.33 (−0.32, 0.97)	0.09 (−0.22, 0.41)	−0.23 (−0.94, 0.48)	0.46 (−0.01, 0.92)	0.23 (<0.01, 0.45)	−0.23 (−0.75, 0.29)

*Results from the final regression models examining associations of opposition-defiance and anxiety symptoms with the three temperamental dimensions and with awakening cortisol in the ADHD and control groups, respectively, as well as the anxiety × group and opposition-defiance × group interaction effects, controlling for sex. ADHD, attention deficit hyperactivity disorder; 95% CI, confidence interval fixed at 95%*.

∧ *p = 0.1*;

** *p < 0.01*.

With the effect of the oppositional-defiant removed in *post-hoc* analyses, the association of inattention with NA in the control and ADHD groups remained non-significant (controls: β = 0.29, 95% CI = −0.22 to 0.81; ADHD: Î^2^ = 0.30, 95% CI = −0.35 t 0.94; both *p* > 0.05). When controlling for oppositional-defiant traits, ratings of hyperactivity-impulsivity remained positively and significantly associated with NA in the controls (β = 0.75, 95% CI = 0.15–1.35, *p* < 0.05) and not significant in the ADHD group (β = 0.12, 95% CI = −0.40 to 0.65, *p* = 0.28). When controlling for anxiety traits, the non-significant association between inattention and NA remained unchanged in both groups (controls: β = 0.33, 95% CI = −0.19 to 0.86; ADHD: Î^2^ = 0.40, 95% CI = −0.25 to 1.04; both *p* > 0.05). Controlling for anxiety traits, hyperactivity-impulsivity remained significantly associated with NA scores in the controls (β = 0.78, 95% CI = 0.17 to 1.38, *p* < 0.05) and not in the ADHD group (β = 0.23, 95% CI = −0.29 to 0.76, *p* = 0.38).

As analyses indicated a specific association of oppositional-defiant and anxiety traits with NA scores only, we did not perform the planned *post-hoc* regressions testing whether the relationships of each ADHD subdomain with the temperament dimensions change when controlling for comorbid traits.

### Do Awakening Cortisol Levels Change When Participants With ADHD Are Under Treatment With Methylphenidate?

For children and adolescents with ADHD who were taking ADHD medications (methylphenidate), cortisol levels did not differ significantly when they were on medication relative to cortisol levels measured after a medication washout for at least 4 days [*F*_(1,63)_ = 1.33; *p* = 0.25]. See **Supplementary Figure S1** for details.

## Discussion

Building on previous hypotheses that link temperament dimensions to biological models (2, 3, 5, 51), we examined temperamental responsiveness, emotionality, and self-regulation in children and adolescents with ADHD and controls, including their relation to a peripheral measure of arousal using salivary cortisol levels. Youths with ADHD demonstrated poorer effortful control and negative emotion regulation skills but no differences in surgency-extraversion relative to controls. Similar associations were found in dimensional analyses for effortful control with ADHD subdimensions (inattention and hyperactivity-impulsivity) but not for negative affectivity. Temperamental emotion regulation difficulty, measured as negative affectivity, was present regardless of comorbid traits such as opposition-defiance and anxiety associated with hyperactivity-impulsivity symptoms. Low awakening cortisol levels were also associated with ADHD diagnosis, but no associations emerged between temperament dimensions and awakening cortisol levels. Atypical levels of temperament traits and a peripheral proxy of arousal levels (i.e., cortisol) may characterize ADHD. However, these properties do not appear to share behavioral and emotional self-regulatory processes in our analysis of a representative clinical sample of children and young people with ADHD.

We found that poorer effortful control and high negative affectivity were significantly associated with ADHD diagnosis. Additionally, low effortful control was associated with both ADHD symptom dimensions (i.e., inattentive and hyperactive-impulsive traits). Further, we observed similar significant or trend-level associations of small-to-moderate effect sizes between high NA and both ADHD symptom dimensions. These findings are consistent with a large body of literature which suggests that children with ADHD display poorer effortful control and negative emotion regulation skills than controls (3, 6–8), and are in line with studies showing an association of these temperamental diatheses with ADHD symptoms in dimensional analyses (9, 12–14, 16, 18, 20). The relationship between hyperactivity-impulsivity and low effortful control scores reached statistical significance in the controls, but not in the ADHD group. While replication in larger samples is needed to confirm these findings, one explanation for these results may be that the mechanisms driving overactive-impulsive behavior in ADHD are not limited to the lack of voluntary self-control measured by EC. As suggested by Nigg et al. (52), the cognitive impairments as well as the emotional lability underlying these traits in ADHD are likely to be multifactorial and further investigations are warranted to disentangle the cognition and the emotion subprofiles that relate to specific clinical features in ADHD. In line with this idea, emotional dysregulation was linked to higher risk for depression and somatoform disorders in a recent study on adults with ADHD (11). In addition, extensive longitudinal studies in adolescents from the general population showed that low effortful control was related to high depressive symptoms (52) and poorer educational attainment (53) in young adulthood. Therefore, as previously suggested (54), further longitudinal studies in clinical populations are warranted to test further whether negative affectivity and poor effortful control detected in childhood and adolescence can help predict adverse outcomes in children with ADHD, ultimately guiding the design of preventative programs.

Despite some suggestive yet non-significant relationships between ADHD symptoms and surgency-extraversion, the lack of group differences in surgency-extraversion traits or a link of these with ADHD symptoms in our data contrasts with the results of previous reports indicating a possible association of surgency-extraversion with ADHD and hyperactivity-impulsivity traits (10, 15, 20). The inconsistency across studies can be viewed in light of the difference in the samples examined. In our study, youths with clinically diagnosed ADHD vs. those with possible ADHD based on parent ratings only (20). Furthermore, the temperament constructs used, that is, the overall surgency-extraversion temperament dimension in our study vs. only subcomponents of surgency and extraversion in the other studies (10, 15). Future studies in large clinical population samples will need to examine further the role of surgency-extraversion in explaining the heterogeneity of ADHD.

Nevertheless, given the relatively lower power in our analyses on the surgency-extraversion subscale, future studies in larger clinical population samples will need to further examine the role of surgency-extraversion in explaining the heterogeneity of ADHD.

In our analyses, temperamental negative affectivity also showed a relationship with opposition-defiant and anxiety traits in participants with ADHD. This is in line with earlier reports of poorer negative emotion regulation skills in children and adolescents with ADHD and an oppositional-defiant disorder (6, 18, 20) or an anxiety disorder (6, 20). Nevertheless, contrary to previous evidence, negative affectivity remained significantly associated with ADHD when controlling for oppositional-defiance (18) and anxiety traits (20), suggesting that negative affectivity may represent a phenotype related to shared risk for several disorders or with symptom domains.

We also found lower awakening cortisol levels in children and adolescents with ADHD compared to controls, confirming many studies demonstrating that low cortisol levels are linked to ADHD (26). Notably, cortisol levels were not affected by the medication status. Indeed, our results align with previous reports that revealed no differences in cortisol levels in children and adolescents with ADHD before and after taking ADHD medications (35, 36). Despite some suggestions that ADHD medications may mitigate the cortisol reductions in ADHD, evidence suggests that awakening cortisol levels return to baseline values after at least 6 months of treatment with methylphenidate (37), which may explain our results. Overall, our finding of reduced cortisol despite ongoing treatment in our study confirms previous hypotheses that suggest a physiological under-arousal in ADHD (33).

Finally, we sought to test the association between cortisol levels and the three main temperament dimensions, in line with the hypothesis that cortisol may be linked to self-regulatory executive, behavioral, and affective processes in ADHD (55). We did not find significant associations between cortisol levels and the surgency-extraversion, negative affect, and effortful control regulation dimensions. To further examine this relationship and confirm that arousal does not share similar underlying mechanisms with temperament in ADHD, studies using repeated measures of cortisol productivity under different active tasks [e.g., such as those used in (30)] and in larger samples are warranted.

Lastly, alternate peripheral markers of arousal that have been linked to validated behavioral measures in ADHD (e.g., the norepinephrine metabolite 3-Methoxy-4-hydroxyphenylglycol; MHPG) [see (56)] should be considered to test the relationship between temperament and ADHD.

The inclusion of a representative sample of youths with ADHD recruited from community services and the investigation of temperament dimensions in combination with a biological measure of arousal are strengths of this study. However, the limitations of this study are worth noting. First, due to the higher prevalence of ADHD in males than in females, our clinical sample included only a small sample of girls with ADHD. Further, we could not measure whether awakening cortisol levels and temperament dimensions differed between boys and girls. Yet, we controlled for sex in all models, limiting the potential effects of sex differences in our analyses.

Moreover, our sample has a wide age range. Stratifying the analyses by age would have resulted in small samples and low power to detect group differences between age groups, limiting our ability to understand whether temperament and peripheral arousal levels may change throughout the developmental stages. However, as temperament profiles have shown stability across life stages (57–59), age effects are unlikely to confound our results. Lastly, our findings should be considered in light of potential overlaps in items on temperament scales and ratings of ADHD symptoms and other psychiatric traits (e.g., activity level, impulsivity, attention focusing, fear, shyness). Previous studies have reported a similar relationship of temperament dimensions with ADHD on scales with overlapping items removed (18). However, future studies should clarify the independence of temperament and symptom ratings using more objective, laboratory-based measures of temperament (60). Stratifying larger samples by other co-occurring psychiatric traits, e.g., the presence of conduct or mood disorders traits (54, 55, 60, 61), may further be considered for testing the relationship of temperament with ADHD (62).

In summary, poor temperamental attention and behavior self-regulation and negative affect were confirmed as features associated with ADHD in a sample of children and young people, regardless of the co-occurrence of symptoms of opposition-defiance and anxiety. Our findings also support the link between ADHD and under-arousal measured as awakening cortisol levels. Nonetheless, suggesting that atypical cortisol function does not share the exact underlying mechanisms with temperamental self-regulatory processes in ADHD. With evidence that early temperamental differences in children with ADHD are linked to the risk of psychiatric comorbidities over time (61, 63), the links of poor negative affect control with ODD and anxiety traits in our study suggest that temperamental profiles could inform early detection programs in ADHD.

## Data Availability Statement

The raw data supporting the conclusions of this article will be made available by the authors, without undue reservation.

## Ethics Statement

The studies involving human participants were reviewed and approved by the Ethical Review Board of the University of Sassari (Ethical approval Study ID Number: 2472/CE; NCT Number NCT04326543). Written informed consent to participate in this study was provided by the participants' legal guardian/next of kin.

## Author Contributions

AC participated the design and conceptualized the study, performed the measurement, collected and interpreted data, performed statistical analysis, and wrote and revised the first version of the manuscript. IV and A-SR contributed to the interpretation of the study results and manuscript revisions. AZ provided advice on the study design and on versions of the manuscript. JK advised on the study design and contributed to the manuscript. SS participated in the study design, study coordination, and manuscript revisions. NA participated in the study design, performed and supervised analyses, contributed to the interpretation of results, contributed to the drafts, and subsequent revisions of the manuscript. All authors read and approved the current version of the manuscript.

## Funding

This study was supported by research funds of the University of Sassari and by the Erasmus Plus (Erasmus+) Programs - Academic Year 2016/2017.

## Conflict of Interest

JK has given talks at educational events sponsored by Medice; all funds are received by King's College London and used for studies of ADHD. AZ served in an advisory or consultancy role for Angelini, EduPharma, Servier. He received conference support or speaker's fee by Angelini and Janssen. He has been involved in clinical trials conducted by Angelini, Janssen, Lundbeck, Otsuka, Roche, Sevier, and Shire. He received royalties from Giunti OS, Oxford University Press. The remaining authors declare that the research was conducted in the absence of any commercial or financial relationships that could be construed as a potential conflict of interest. The reviewer PK declared a shared affiliation with the A-SR at the time of review.

## Publisher's Note

All claims expressed in this article are solely those of the authors and do not necessarily represent those of their affiliated organizations, or those of the publisher, the editors and the reviewers. Any product that may be evaluated in this article, or claim that may be made by its manufacturer, is not guaranteed or endorsed by the publisher.
