# Supplementary figures and images for: Temperament Dimensions and Awakening Cortisol Levels in Attention-Deficit/Hyperactivity Disorder
Source: Front Psychiatry. 2022 Apr 25;13:803001. doi: 10.3389/fpsyt.2022.803001 (PMC9081759; doi:10.3389/fpsyt.2022.803001)

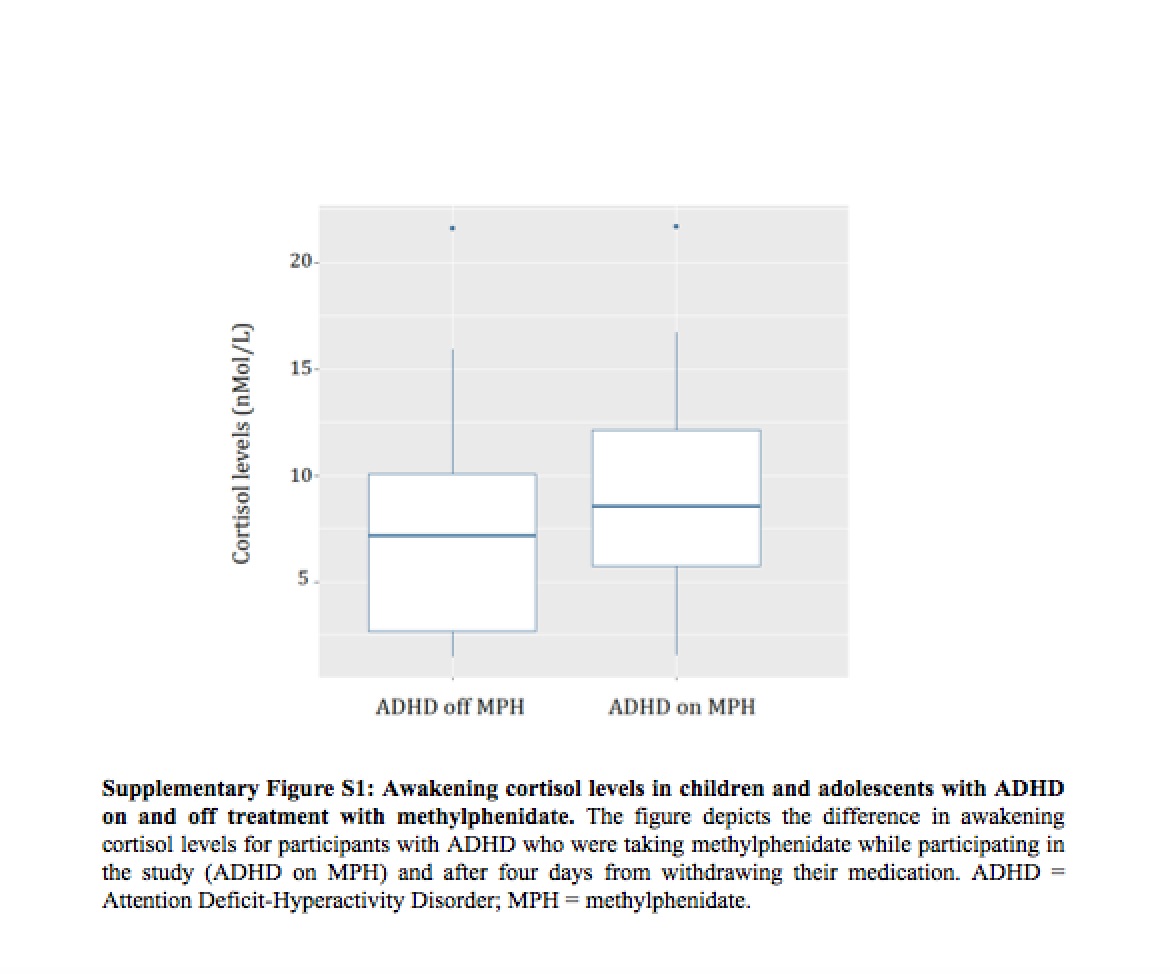

Supplement: Supplementary file 1 [file Image_1.jpeg]
