# Supplementary material for: Temperament Dimensions and Awakening Cortisol Levels in Attention-Deficit/Hyperactivity Disorder
Source: Front Psychiatry. 2022 Apr 25;13:803001. doi: 10.3389/fpsyt.2022.803001 (PMC9081759; doi:10.3389/fpsyt.2022.803001)
Supplement: Supplementary file 2 [file Table_1.docx]

**Supplementary Table 1. Indices of reliability for psychometric measures**

|  | Corrected Item-  Total Correlation | Cronbach's Alpha | |  |
| --- | --- | --- | --- | --- |
| **Total CPRS:RL ODD** | 0.77 | | 0.65 | |
| **Total CPRS:RL ANX** | 0.61 | | 0.66 | |
| **Effortful control** | -0.61 | | 0.77 | |
| **Surgency-extravertion** | 0.03 | | 0.76 | |
| **Negative affectivity** | 0.44 | | 0.75 | |
| **SNAP- In** | 0.82 | | 0.62 | |
| **SNAP- Hy** | 0.78 | | 0.62 | |

Cronbach’ s Alpha value > 0,9 = Excellent; > 0,8 = Good; > 0,7 Acceptable; > 0,6 Questionable; > 0,5Poor;

< 0,5 Unacceptable (George and Mallery, 2003).

We calculated the reliability test for metrical variables, based on our psychometric data observations: the

three board temperament effortful control (EC), surgency-extraversion (SE) and negative affect (NA)

scales, the two SNAP-IV subscales for the inattention and hyperactivity-impulsivity symptoms and the two scales

of the CPRS-R:L for opposition defiant (ODD) and anxiety (ANX) traits.
